# Supplementary material for: The “Neural Shift” of Sleep Quality and Cognitive Aging: A Resting-State MEG Study of Transient Neural Dynamics
Source: Front Aging Neurosci. 2022 Jan 31;13:746236. doi: 10.3389/fnagi.2021.746236 (PMC8842663; doi:10.3389/fnagi.2021.746236)
Supplement: Supplementary file 1 [file Data_Sheet_1.docx]

**Supplementary Materials**

|  |  |
| --- | --- |
| 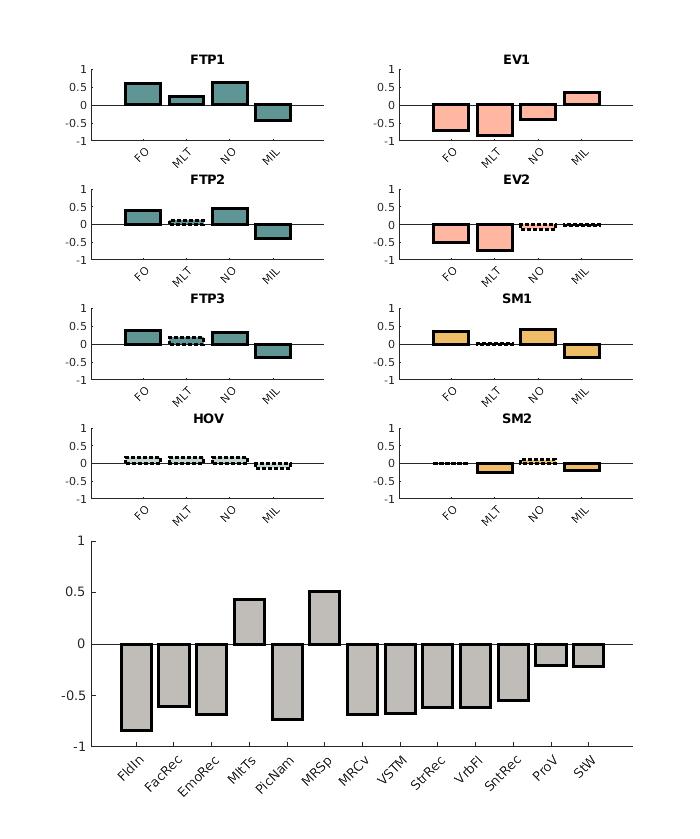 | 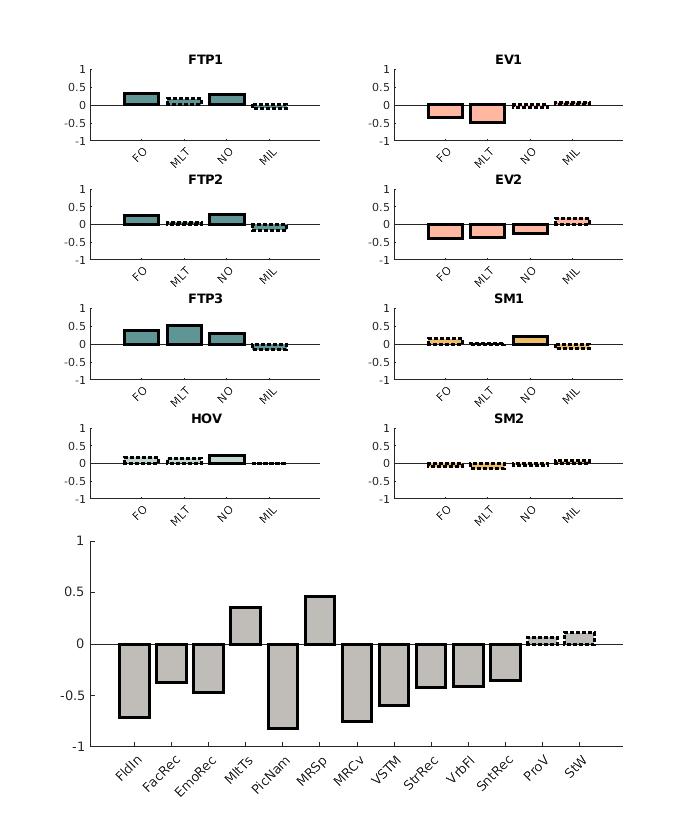 |

**Supplementary Figure 1.** Loadings obtained via the analysis relating brain network (HMM) measures with cognitive measures using PLS (Panel A) and CCA (Panel B). Overall, the results are highly similar. The CCA results displayed in Panel B were obtained using the same method reported in Tibon et al. (2021), but on a subset of the sample (with N=564 instead of N=594) due to the exclusion of participants with no sleep measures.

**Moderation analysis with additional covariates**

In order to ensure that the relations between the HMM profile and the sleep profile exist beyond potential covariates, we repeated the moderation analysis with sex and session time (i.e., the time of the day on which the data were collected) as additional predictors. Thus, the model included participants’ scores for the HMM profile, age, the interaction between HMM profile and age, sex, and session time as predictors, and participants’ scores for the sleep profile as the dependent variable.

The results did not change in a meaningful way i.e. the HMM scores were significantly associated with sleep scores (β = .06, *t*(549) = 2.7, *p* = .006), and so was age (β = -.28, *t*(549) = -5.3, *p* < .001). Furthermore, the analysis revealed a significant effect of sex (β = -.19, *t*(549) = -3.5, *p* < .001).
